# High genetic diversity and strong genetic structure of *Strongyllodes variegatus* populations in oilseed rape production areas of China

**DOI:** 10.1186/s12862-021-01752-6

**Published:** 2021-02-09

**Authors:** Hai-Xia Zhan, Zhong-Ping Hao, Rui Tang, Li-Ni Zhu, Jing-Jiang Zhou, Shu-Min Hou

**Affiliations:** 1 National Oil Crops Improvement Center, Hefei Rapeseed Subcenter, Crop Research Institute, Anhui Academy of Agricultural Sciences, Hefei, 230031 China; 2 Guangdong Key Laboratory of Animal Conservation and Resource Utilization, Guangdong Public Laboratory of Wild Animal Conservation and Utilization, Institute of Zoology, Guangdong Academy of Sciences, Guangzhou, 510260 China; 3 Gansu Biocontrol Engineering Laboratory of Crop Diseases and Pests, Gansu Agricultural University, Lanzhou, 730070 China

**Keywords:** Gene flow, Genetic differentiation, Haplotype, Oilseed rape, Population genetic pattern, *Strongyllodes variegatus*

## Abstract

**Background:**

*Strongyllodes variegatus* (Fairmaire) is a major insect pest of oilseed rape in China. Despite its economic importance, the contribution of its population genetics in the development of any suitable protection control strategy for the management of oilseed rape crops is poorly studied. It is a much urgent need to prevent its spread to the rest of the world.

**Results:**

Using the sequences of mitochondrial DNA cytochrome *c* oxidase subunit I (*COI*) and cytochrome *b* (*Cytb*) as genetic markers, we analyzed the population genetic diversity and structure of 437 individuals collected from 15 *S. variegatus* populations located in different oilseed rape production areas in China. In addition, we estimated the demographic history using neutrality test and mismatch distribution analysis. The high level of genetic diversity was detected among the *COI* and *Cytb* sequences of *S. variegatus*. The population structure analyses strongly suggested three distinct genetic and geographical regions in China with limited gene flow. The Mantel test showed that the genetic distance was greatly influenced by the geographical distance. The demographic analyses showed that *S. variegatus* had experienced population fluctuation during the Pleistocene Epoch, which was likely to be related to the climatic changes.

**Conclusion:**

Overall, these results demonstrate that the strong genetic structure of *S. variegatus* populations in China, which is attributed by the isolation through the geographical distance among populations, their weak flight capacity and subsequent adaptation to the regional ecological conditions.

## Background

The brown beetle, *Strongyllodes variegatus* (Fairmaire) (Coleoptera: Nitidulidae), feeds on brassicaceous plant species [1, 2], which often co-occurs with the pollen beetle, *Meligethes aeneus* [3]. The *S. variegatus* adults chew up flowers, buds and leaves, and create crescent-shaped bites where the mature females lay eggs. After hatching, the larvae feed on mesophyll resulting in irregular bubble-shaped wounds before pupation in soil. The wounded leaves become necrotic and abscise prematurely [4, 5]. Recently, the leaf damage of oilseed rape crops by this beetle has become more and more serious, so that it has become a major insect pest of oilseed rape crops. In spring 2013, *S. variegatus* population broke out in Hanshan, Anhui province, destructing 97% of oilseed rape leaves [6].

*S. variegatus* displays specific ecological characteristics to temperature and photoperiod of geographical regions. In spring oilseed rape areas, it reproduces once or twice a year [2]. However, only two generations occur in winter oilseed rape areas [6]. In addition, *S. variegatus* has a high reproductive ability [4] and can fly 30 ~ 40 m in 2 min [2]. In Anhui, the overwintering adults begin to appear in March. When the temperature is more than 30 ℃, the adults stay in soil in summer, and some of them are mixed into the harvested rapeseed. They appear on cruciferous vegetables in September, then move to rape fields and cause damages in October. When the temperature is low in November, they move back to soil and overwinter in soil [4, 6].

*S. variegatus* is generally distributed in the middle and lower reaches of the Yangtze River valley. It was first found on the spring oilseed rape plants in Ningxia, Gansu province, China in 1993 [2], and then on the winter oilseed rape crops in Hanshan, Anhui province in 2008 [4]. For the past few years, we investigated oilseed rape production areas in China and found that this pest has spread to Chongqing municipality and Qinghai, Gansu, Sichuan, Shaanxi, Hubei, Anhui and Jiangsu provinces (unpublished). Currently, it is widely distributed around China but has not yet been found globally in the rest of the world except in China. The phylogeography and population genetics of *S. variegatus* have not been studied. Consequently, it is in urgent demand to conduct the genetics studies and understand the genetic diversity and structure of *S. variegatus* populations in order to manage and control this pest.

Population genetic studies on crop pests can provide information on the spatial scales at which population structure is established and gene flow occurs. Such information can be used in defining relevant strategies for pest control [7]. In addition, genetic diversity contains the information on past and present demography that could be useful to characterize the demographic history of crop pests [8]. In recent years, more and more molecular markers have been used to study insect population genetics, demonstrating the importance of phylogeographical approaches [9]. The insect molecular markers mainly include the sequences of nuclear DNA and mitochondrial DNA (mtDNA). Mitochondrial genes have a faster evolution rate than nuclear genes, and are more informative for studying phylogenetic evolution, especially the degree of inter- and intra-specific population differentiation and the level of gene flow [9, 10]. Thus, the fragments of the mtDNA cytochrome *c* oxidase subunit I (*COI*) and cytochrome b (*Cytb*) have been widely used among insect molecular markers to study population genetic variation and differentiation of insects, for example, *Dendrolimus kikuchii*, *Chilo suppressalis* and *Agriosphodrus dohrni* [11–15]. The *COI* and *Cytb* genes were also used to track the colonization routes of *Halyomorpha halys* and to identify the places where the insect has originated [16–18].

In this study, we use *COI* and *Cytb* genes to elucidate for the first time the genetic diversity and structure of 15 *S. variegatus* populations occurring on the oilseed rape production areas in China. We hypothesize that the populations would have a high level of genetic diversity and a clear genetic structure. At the same time, the efficient molecular data collected are used to assess if historical geographic events and associated ecological adaptations had played an important part in shaping the observed genetic and geographic patterns of this pest in China.

## Results

### Genetic variation of *S. variegatus* populations

Seventy haplotypes of the *COI* gene and 67 haplotypes of the *Cytb* gene were identified from the 15 populations. The *S. variegatus COI* fragment (652 bp) and *Cytb* fragment (421 bp) have 45 (6.9%) and 40 (9.5%) variable sites with 28 and 23 parsimony informative sites, respectively (Table 1). The base composition of the two genes is adenine (A) and thymine (T) (67.5% and 73.3%, respectively) biased, which is common for insect mitochondrial genes. The haplotype diversity (*Hd*) ranges from 0.424 to 0.913 (mean = 0.865) and the nucleotide diversity (*π*) ranges from 0.00072 to 0.00462 (mean = 0.00427) for the *COI* gene (Table 1). Similarly, the *Hd* ranges from 0.464 to 0.833 (mean = 0.834) and *π* ranges from 0.00119 to 0.00539 (mean = 0.00479) for the *Cytb* gene (Table 1).

**Table 1:** Genetic diversity indices and neutrality test for mitochondrial *COI* and *Cytb* markers in all analyzed *Strongyllodes variegatus* populations

Marker	Population code	Region^a^	*N*	*S*	*Hn*	*Hd*	*π*	*k*	Tajima's *D*	*P*	Fu's *Fs*	*P*
*COI*	GDQH		34	4	5	0.702	0.00166	1.082	0.263	*NS*	− 0.286	*NS*
	HZGS		34	8	9	0.702	0.00192	1.255	− 1.059	*NS*	− 3.893	**
	ZYGS		24	5	6	0.649	0.00121	0.790	− 1.188	*NS*	− 2.707	*
	GYSC		30	14	14	0.855	0.00378	2.467	− 1.008	*NS*	− 6.799	***
	HZSX		30	14	15	0.913	0.00462	3.018	− 0.487	*NS*	− 6.672	***
	AKSX		35	13	13	0.852	0.00431	2.810	− 0.351	*NS*	− 3.961	*
	FJCQ		30	11	14	0.857	0.00328	2.138	− 0.740	*NS*	− 7.898	***
	ESHB		8	6	6	0.893	0.00257	1.679	− 1.280	*NS*	− 3.114	**
	LCHB		32	9	11	0.764	0.00320	2.085	− 0.206	*NS*	− 3.819	*
	AQAH		37	13	11	0.580	0.00146	0.949	− 2.201	***	− 8.187	***
	LAAH		21	4	5	0.424	0.00072	0.467	− 1.654	*	− 3.127	***
	HFAH		34	6	6	0.574	0.00119	0.775	− 1.306	*NS*	− 2.271	*NS*
	CHAH		26	5	6	0.520	0.00092	0.597	− 1.543	*	− 3.524	***
	NJJS		31	5	4	0.458	0.00095	0.619	− 1.367	*NS*	− 0.697	*NS*
	ZJJS		31	5	6	0.628	0.00118	0.770	− 1.041	*NS*	− 2.417	*
		NW	92	14	15	0.713	0.00183	1.193	− 1.565	*	− 9.255	***
		CC	165	30	43	0.856	0.00397	2.587	− 1.471	*	− 26.732	***
		CE	80	20	20	0.544	0.00113	0.736	− 2.132	**	− 21.274	***
	Total		337	45	70	0.865	0.00427	2.786	− 1.628	*	− 25.887	***
*Cytb*	GDQH		34	6	6	0.708	0.00417	1.758	0.547	*NS*	0.186	*NS*
	HZGS		34	4	4	0.469	0.00285	1.198	0.558	*NS*	1.002	*NS*
	ZYGS		24	3	4	0.583	0.00161	0.678	− 0.394	*NS*	− 0.714	*NS*
	GYSC		30	14	14	0.833	0.00539	2.271	− 1.192	*NS*	− 7.424	***
	HZSX		30	11	11	0.832	0.00472	1.986	− 0.916	*NS*	− 4.300	*
	AKSX		35	13	13	0.810	0.00455	1.916	− 1.258	*NS*	− 6.437	**
	FJCQ		30	9	10	0.791	0.00300	1.262	− 1.381	*NS*	− 5.530	***
	ESHB		8	2	3	0.464	0.00119	0.500	− 1.310	*NS*	− 0.999	*NS*
	LCHB		32	18	15	0.752	0.00359	1.51	− 2.252	***	− 12.320	***
	AQAH		37	8	9	0.718	0.00255	1.075	− 1.273	*NS*	− 4.442	**
	LAAH		21	3	4	0.71	0.00215	0.905	0.223	*NS*	− 0.187	*NS*
	HFAH		34	9	9	0.784	0.00251	1.239	− 1.322	*NS*	− 3.954	**
	CHAH		26	7	9	0.726	0.00272	1.145	− 1.151	*NS*	− 5.076	***
	NJJS		31	9	9	0.776	0.00347	1.462	− 1.082	*NS*	− 3.413	*
	ZJJS		31	8	9	0.697	0.00215	0.903	− 1.655	*	− 5.812	**
		NW	92	9	9	0.638	0.00354	1.492	− 0.393	*NS*	− 1.395	*NS*
		CC	165	33	43	0.826	0.00436	1.837	− 1.992	**	− 27.537	***
		CE	80	20	24	0.741	0.00276	1.162	− 1.799	*	− 21.480	***
	Total			40	67	0.834	0.00479	2.015	− 1.819	**	− 26.759	***
*COI* + *Cytb*		NW	92	23	23	0.800	0.00250	2.686	− 1.208	*NS*	− 11.042	***
		CC	165	63	82	0.957	0.00412	4.423	− 1.847	**	− 25.523	***
		CE	80	40	48	0.881	0.00177	1.898	− 2.139	**	− 27.540	***

For each population, the number of individuals (*N*), the number of variable sites (*S*), number of haplotypes (*Hn*), haplotype diversity (*Hd*), nucleotide diversity (*π*), average number of nucleotide differences (*k*) and Tajima's *D* and Fu's *Fs* test statistics for selective neutrality are given

Values are significant at *P ≤ 0.05; **P ≤ 0.01; ***P ≤ 0.001; *NS*, not significant

**Fig. 1:**
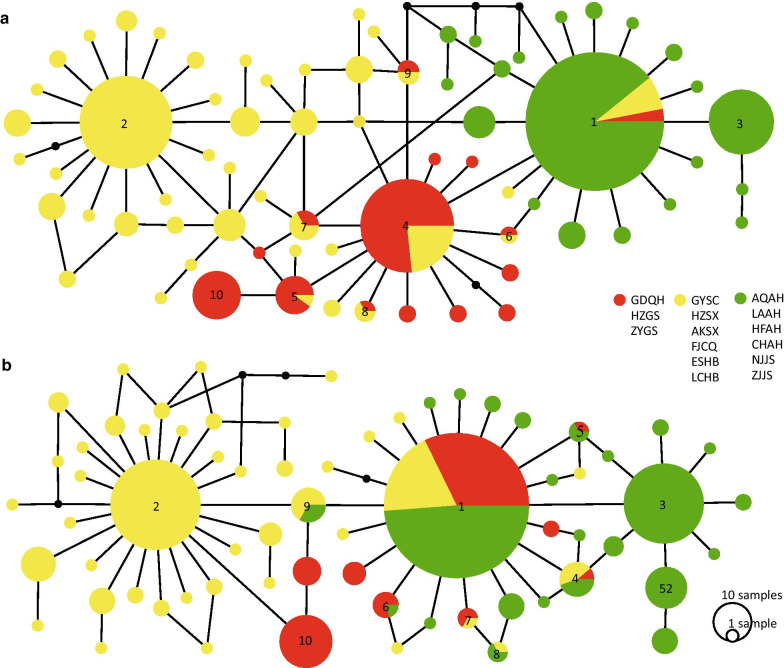
Haplotype networks estimated from the sequences of (**a**) the *COI* gene and (**b**) the *Cytb* gene. The circles represent haplotype, the numbers in the circle represent name of haplotype, the small black circles represent missing haplotypes that were not observed, the circle size denotes the total haplotype frequency, while each slice represents the haplotype frequency in different populations, and the lines between linked haplotypes correspond to one mutation. Three haplotype regions are indicated by three different colors: the NW region (red), the CC region (yellow) and the CE region (green). Fifteen populations include GDQH (Guide, Qinghai province), HZGS (Hezheng, Gansu province), ZYGS (Zhenyuan, Gansu province), GYSC (Guangyuan, Sichuan province), HZSX (Hanzhong, Shaanxi province), AKSX (Ankang, Shaanxi province), FJCQ (Fengjie, Chongqing municipality), ESHB (Enshi, Hubei province), LCHB (Lichuang, Hubei province), AQAH (Anqing, Anhui province), LAAH (Liu'an, Anhui province), HFAH (Hefei, Anhui province), CHAH (Caohu, Anhui province), NJJS (Nanjing, Jiangsu province), and ZJJS (Zhenjiang, Jiangsu province)

^a^Regions as defined in Fig. 1

### Haplotype analyses of the *COI* and *Cytb* genes

The distribution of the haplotypes for the two genes across the populations studied is shown in Additional file 1: Table S1. The rarefaction analyses showed that the curves converged on an asymptote (Additional file 2: Fig. S1). The *COI* haplotypes (H1-H70) included 34 (48.6%) unique haplotypes (Additional file 1: Table S2). The four most frequent haplotypes (H1-H4) were found in 132 (30.2%), 59 (13.5%), 29 (6.6%), and 60 (13.7%) individuals (Additional file 1: Table S2; Fig. 1a). The haplotype 1 (H1) was in almost all populations except the populations from GDQH, FJCQ and ESHB, whereas the haplotype 2 (H2) was only in the populations from GYSC, HZSX, AKSX, FJCQ, ESHB and LCHB (Additional file 1: Table S2). The *Cytb* haplotypes (H1-H67) had 35 (52.2%) unique haplotypes, among which 32 were observed in more than one individual (Additional file 1: Table S2). Three most frequent haplotypes (H1-H3) were found in 158 (36.2%), 61(14.0%) and 48 (10.9%) individuals (Additional file 1: Table S2; Fig. 1b). The haplotype 1 (H1) was found in all populations except ESHB population, whereas the haplotype 3 (H3) was only discovered in the populations from AQAH, LAAH, HFAH, CHAH, NJJS and ZJJS (Additional file 1: Table S1).

The haplotype distribution and haplotype network analyses (see below) of both *COI* and *Cytb* genes revealed that *S. variegatus* populations could be divided into three major geographical distribution regions or haplogroups: the northwestern China (NW) haplogroup (GDQH, HZGS and ZYGS populations), the central China (CC) haplogroup (GYSC, HZSX, AKSX, FJCQ, ESHB and LCHB populations) and the central and eastern China (CE) haplogroup (AQAH, LAAH, HFAH, CHAH, NJJS and ZJJS populations).

For the haplotype network of the *COI* gene, there was only one common haplotype (H1) in three haplogroups. The haplotype 2 (H2) was only detected and abundant in the CC haplogroup. The haplotype 3 (H3) was only discovered in the CE haplogroup. There were six common haplotypes (H4-H9) between the NW haplogroup and the CC haplogroup. A total of five missing haplotypes was observed in all populations (Fig. 1a). Similarly, for the haplotype network of the *Cytb* gene, there were two common haplotypes (H1, H4) in three haplogroups. The haplotype 2 (H2) was most abundant and only detected in the CC haplogroup. The haplotype 3 (H3) was only discovered in the CE haplogroup. The haplotypes 5–6, 7, 8–9 (H5–H6, H7, H8–H9) were common in the NW and the CC haplogroups, the NW and the CE haplogroup, the CC and the CE haplogroup, respectively. A total of four missing haplotypes was observed in the CC haplogroup (Fig. 1b).

### Population genetic differentiation

A strong genetic divergence was observed across populations (*F*_*ST*_ = 0.425, *P* < 0.0001, Table 2). The *F*_*CT*_ value among three regions (NW, CC and CE) was highly significant (*F*_*CT*_ = 0.470, *P* < 0.0001, Table 2), further demonstrating that *S. variegatus* populations in China is divided into three regions. A significant genetic differentiation was observed among populations within the regions (*F*_*SC*_ = 0.072, *P* < 0.0001, Table 2), and within the populations (*F*_*ST*_ = 0.508, *P* < 0.0001, Table 2) based on the combined data of the *COI* and *Cytb* genes. The percentages of genetic variation within the populations (60.16% in the populations between NW and CC regions, and 56.00% in the populations between NW and CE regions) were significantly higher than those of the comparisons between the regions (33.89% between NW and CC regions, 33.88% between NW and CE regions) (Table 2). However, the percentage of genetic variations between CC and CE regions (54.95%) was higher than 42.82% within the populations (Table 2), indicating that there is limited gene flow between the CC and CE regions.

**Table 2:** Hierarchical analysis of molecular variance (AMOVA) in collected *Strongyllodes variegatus* samples from 15 populations

Source of variation	*df*	Sum of squares	% of variation	Fixation indices
All populations				
Among populations	14	446.669	42.50	
Within populations	422	599.926	57.50	*F*_*ST*_ = 0.425***
Three regions				
Among regions	2	391.765	47.01	*F*_*CT*_ = 0.470***
Among populations within regions	12	54.904	3.80	*F*_*SC*_ = 0.072***
Within populations	422	599.926	49.18	*F*_*ST*_ = 0.508***
NW vs. CC				
Among regions	1	124.847	33.89	*F*_*CT*_ = 0.339**
Among populations within regions	7	46.483	5.95	*F*_*SC*_ = 0.090***
Within populations	248	438.452	60.16	*F*_*ST*_ = 0.398***
NW vs. CE				
Among regions	1	89.300	38.88	*F*_*CT*_ = 0.389**
Among populations within regions	7	26.418	5.11	*F*_*SC*_ = 0.084***
Within populations	263	265.672	56.00	*F*_*ST*_ = 0.440***
CC vs. CE				
Among regions	1	332.830	54.95	*F*_*CT*_ = 0.550***
Among populations within regions	10	36.907	2.23	*F*_*SC*_ = 0.050***
Within populations	333	495.727	42.82	*F*_*ST*_ = 0.572***

AMOVA partitioned among all populations and three regions: NW region (GDQH, HZGS, ZYGS), CC region (GYSC, HZSX, AKSX, FJCQ, ESHB, LCHB) and CE region (AQAH, LAAH, HFAH, CHAH, NJJS, ZJJS)

^**^P ≤ 0.001, ***P ≤ 0.0001 after 1023 permutations

The pairwise *F*_*ST*_ values based on the combined date of the *COI* and *Cytb* genes among populations ranged from − 0.015 to 0.811 (Table 3). In 105 comparisons, 88 comparisons showed a significantly higher genetic differentiation. The pairwise *F*_*ST*_ values among populations within the CC and CE regions were less than 0.159, while the pairwise *F*_*ST*_ values between the populations from CC and CE regions were above 0.409. In addition, the pairwise *F*_*ST*_ values were highly significant among the regions (*F*_*ST*_ > 0.25, *P* < 0.001, Table 4), and the gene flow among the regions was estimated extremely low (*Nm* < 1, Table 4), suggesting a limited gene flow among the regions. The results are greatly consistent with those obtained by the analysis of molecular variance (AMOVA) described in above sections.

**Table 3:** Pairwise *F*_*ST*_ values among populations of *Strongyllodes variegatus* based on the combined data of the *COI* and *Cytb* genes

	GDQH	ZYGS	HZGS	GYSC	HZSX	AKSX	FJCQ	ESHB	LCHB	AQAH	LAAH	HFAH	CHAH	NJJS	ZJJS
GDQH															
ZYGS	**0.086**														
HZGS	**0.330**	**0.124**													
GYSC	**0.400**	**0.438**	**0.533**												
HZSX	**0.249**	**0.230**	**0.326**	**0.066**											
AKSX	**0.240**	**0.226**	**0.325**	**0.078**	− 0.015										
FJCQ	**0.454**	**0.488**	**0.597**	**0.025**	**0.089**	**0.077**									
ESHB	**0.573**	**0.627**	**0.767**	0.024	**0.159**	**0.161**	0.009								
LCHB	**0.443**	**0.489**	**0.598**	0.008	**0.103**	**0.096**	0.006	0.004							
AQAH	**0.477**	**0.369**	**0.336**	**0.583**	**0.431**	**0.434**	**0.644**	**0.756**	**0.642**						
LAAH	**0.534**	**0.458**	**0.489**	**0.593**	**0.454**	**0.460**	**0.669**	**0.811**	**0.661**	**0.074**					
HFAH	**0.478**	**0.378**	**0.363**	**0.574**	**0.423**	**0.427**	**0.639**	**0.752**	**0.635**	− 0.002	**0.071**				
CHAH	**0.482**	**0.377**	**0.366**	**0.566**	**0.409**	**0.414**	**0.638**	**0.771**	**0.633**	− 0.014	**0.088**	0.000			
NJJS	**0.492**	**0.399**	**0.387**	**0.575**	**0.432**	**0.436**	**0.643**	**0.754**	**0.638**	0.019	0.040	− 0.001	0.027		
ZJJS	**0.495**	**0.395**	**0.388**	**0.583**	**0.430**	**0.434**	**0.651**	**0.779**	**0.646**	0.015	**0.152**	− 0.003	0.019	0.026	

Significant *F*_*ST*_ values are shown in bold (*P* = 0.05)

**Table 4:** Pairwise *F*_*ST*_ values (below diagonal) and gene flow (above diagonal) pairwise and within the geographical regions based on the combined data of the *COI* and *Cytb* genes

Regions^a^	–	NW	CC	CE
–	–	1.131 ^b^	3.917	9.009
NW	0.181		0.457	0.373
CC	0.060	0.354 ***		0.202
CE	0.027	0.401 ***	0.553 ***	

^a^Regional abbreviations are defined in Fig. 1 and Table 2

^b^Gene flow (Nm) was calculated from *Fst* as: Nm = (1−*Fst*) / 4 *Fst*

^***^P < 0.001

**Fig. 2:**
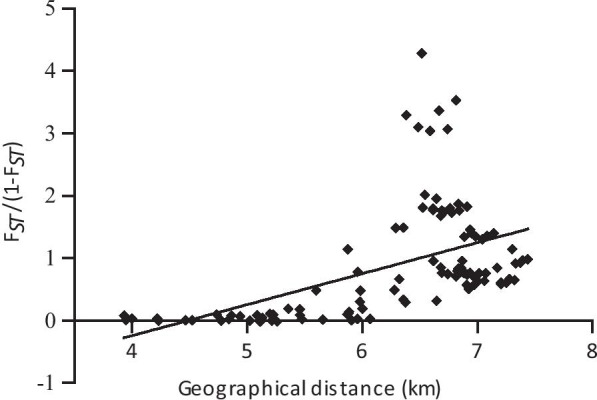
Scatter plots of genetic divergence against geographical distance. The genetic divergence *F*_*ST*_/(1 − *F*_*ST*_) and the geographic distance (ln) were compared using the Mantel test with 10,000 permutations. There is a strong correlation between the genetic divergence and the geographical distance in the pairwise comparisons of all populations (r = 0.500, *P* < 0.0001)

The Mantel test based on the combined data of the *COI* and *Cytb* genes revealed a significant correlation between the genetic distance (*F*_*ST*_/(1 − *F*_*ST*_)) and the geographical distances among all populations (*r* = 0.500, *P* < 0.0001, Fig. 2).

### Demographic analyses

**Fig. 3:**
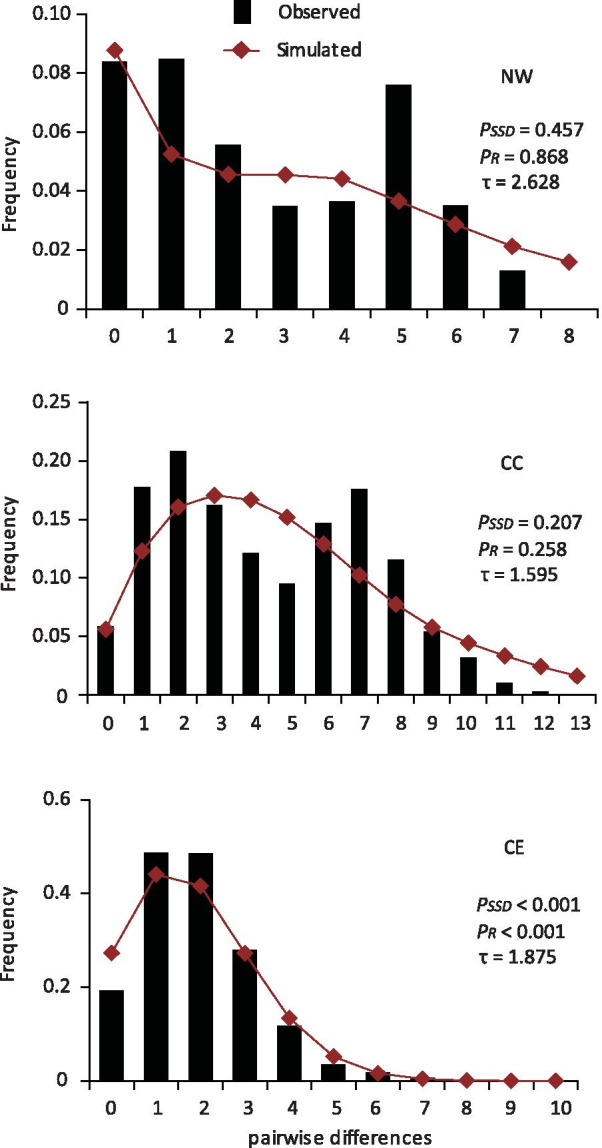
Pairwise mismatch distributions based on the combined data of the *COI* and *Cytb* genes for three derived regions. The x coordinate represents the number of pairwise differences among sequences, and the y coordinate represents the frequencies of pairwise differences in each region. The significance values (*P*) of the parameters were evaluated with 1000 simulations; *P*_SSD_: *P* value for SSD (sum of squared deviations) *P*_R_: *P* value for Rag (Harpending’s raggedness index); τ: the index of population expansion

The Tajima’s *D* values obtained with either single or combined data of the two genes in the NW region were negative, but not significant (*P* > 0.05, Table 1). The Tajima’s *D* and Fu’s *Fs* values in the CC and CE regions were negative and highly significant (*P* < 0.05, Table 1), whereas the CE region showed significant sum of squares deviation (SSD) values (*P* < 0.05, Fig. 3, Additional file 3: Fig. S2). Thus, for the NW and CE regions, the sudden expansion hypothesis was rejected. However, the distributions of the pairwise differences obtained with single and combined gene data in the CC region were unimodal with non-significant SSD and Harpending’s raggedness index (Rag) values (Fig. 3, Additional file 3: Fig. S2), suggesting an expansion event in the CC region. The tau values (*τ*), a rough estimate of the population expansion, were approximately 3.842 (*COI* data), 2.016 (*Cytb* data), and 1.595 (*COI* + *Cytb* data) mutation units for the CC region. For the NW and CE regions, *τ* was 1.344 and 0.766 in the data of the *COI* gene, 3.693 and 0.875 in the data of the *Cytb* gene, and 2.628 and 1.875 in the combined data of the *COI* and *Cytb* genes (Fig. 3, Additional file 3: Fig. S2).

## Discussion

Using two mitochondrial genes, we investigated the genetic diversity and structure of 437 individuals collected from 15 *S. variegatus* populations from different oilseed rape production areas in China. The results exhibited a high genetic diversity and clear genetic structure of *S. variegatus* populations in China.

Based on the analyses of the mtDNA sequences, haplotype distribution, haplotype networks and AMOVA, three genetically diverse and geographically distinct regions of *S. variegatus* distribution in China are classified, namely the northwestern China (NW) region, the central China (CC) region, and the central and eastern China (CE) region. A high proportion of total genetic variance is attributed to the variations within the populations (49.18%) and among the regions (47.01%). This indicates that the largest source of variation might not be due to the geographical barriers among the regions but to the variations among individuals within the populations. It was reported previously that the variations among individuals within the populations had a significant effect on the genetic structure of *Chilo suppressalis* [19]. This contrasts with the studies of *Myotis myotis* and *Plecotus austriacus* [20, 21], which showed that the geographical barrier was the most important effect. Other factors could also play a significant role on the genetic structure. Chen and Dorn analyzed the genetic variation of *Cydia pomonella* populations in Switzerland and found that host specificity, geographic isolation, intrinsic flight capacity and anthropogenic measures could all shape the population structure [22].

A limited gene flow (*Nm* < 1) was revealed among the regions by the current study. It is known that once populations have become genetically differentiated, their genetic divergence status can be maintained if they have differentially adapted to regional ecological conditions, since geographic variation in selection can act as a strong barrier to gene flow [23]. Our analysis also suggested a large gene flow among the populations within the CC and CE regions. This may be due to the geographical isolation as the Mantel test results showed that the gene flow between the populations was greatly influenced by geographical distance. This strong isolation-by-distance relationship in our study may be also due to the limited flight capacity of *S. variegatus*. It was reported that *S. variegatus* can fly 30 ~ 40 m in 2 min [2]. However, the flight ability of *S. variegatus* is less than tens of kilometres and would not be enough to weaken the isolation-by-distance relationships and to increase the potential for allopatric or parapatric speciation [24, 25]. On the other hand, the three regions shared common haplotypes, suggesting small amounts of gene flow among the regions. This may be because some of adults are mixed into the harvested rapeseed over summer [4, 6]. Human intervention in the method of alternating seed breeding in a different location of oilseed rape crops could also play an important role in the mixing of populations from distant geographic regions and provide the conditions for the gene flow among the regions [6].

Gene flow in insects has been reported to increase with mobility, which is more pronounced on herbaceous plants, and this feature is strong especially in agricultural pests [26]. The large genetic variation within populations was also found for the pollen beetle, *Meligethes aeneus*, another oilseed rape pest [9, 27–29]. However, no population structure of the pollen beetle could be found in five provinces of Sweden [28]. *M. aeneus* is found to have high altitude flights (up to ca 200 m) at specific points during the year and low-altitude flights at multiple periods [29], which could help to disperse over large distances with the assistance of prevailing wind currents [30], resulting in the high gene flow similar to the diamondback moths, *Plutella xylostella* [31].

Both the neutrality test and the mismatch distribution analysis indicated a population expansion in the CC region. Furthermore, the phylogeographic patterns of the *COI* and *Cytb* haplotype networks are roughly composed of three “star-like” clusters. Based on 2.3% per site per million years [32], the expansion time of the CC region for the *COI* gene and *Cytb* gene can be estimated to be 104 and 128 ka years ago, respectively, within the interglacial time of the Pleistocene. Vast glaciers developed at that time in Tibetan Plateau, Qinling Mountain and even in the Yangtze River valley [33, 34], which could trigger episodes of range contractions and expansions in many plant and animal species [35–37].

In China, the management practices against *S. variegatus* have primarily focused on using chemicals. The investigation of the genetic diversity of *S. variegatus* populations can provide a useful guide for controlling this pest. Furthermore, localized populations with similar genetic structure should be considered as a same management unit for most effective control [38]. For isolated populations, various management methods should be used, especially, a variety of chemical pesticides with different properties and modes of action. Additional research will be carried out using other molecular markers, such as nuclear genes, or even faster evolutionary markers, such as microsatellites to obtain better understanding of the population genetic structure and evolutionary history of *S. variegatus* in China, and in the rest of the world if the pest would occur in future.

## Conclusions

The current study provides the first population genetic analysis of *S. variegatus*, a serious pest of oilseed rape crops. The high variability observed using the *COI* and *Cytb* molecular markers indicates that the markers are useful for measuring the genetic patterns in *S. variegatus* populations. The distinct distribution of *S. variegatus* populations in China could be divided into three genetic haplogroups and geographical regions with the limited gene flow among them. The distribution of this species in oilseed rape production areas in China is mainly structured by the isolation through geographical distance among the populations and their weak flight capacity. The population expansion signature in the CC region might be related to the climatic changes during the Pleistocene. The phylogenetic information obtained from this study could be used to guide the development of suitable protection control strategies against the insect pests of oilseed rape crops.

## Methods

### Sampling

A total of 437 *S. variegatus* individuals was collected from 15 populations in China (Additional file 1: Table S2). The sample sizes ranged from 24 to 37 individuals per population except eight individuals for the ESHB population (Additional file 1: Table S2). All *S. variegatus* individuals were freshly collected from the fields and immediately stored in absolute ethyl ethanol at -20℃ before molecular analysis.

### DNA extraction, amplification, and sequencing

Total genomic DNA was extracted from each *S. variegatus* specimens following the DNeasy Blood & Tissue Kit protocol (QIAGEN, Germany). The primers used were LCO-1490 (5′-GGTCAACAAATCATAAAGATATTGG-3′) and HCO-2198 (5′-TAAACTTCAGGGTGACCAAAAAATCA-3′) for the regions of the *COI* gene and CB1 (5′-TATGTACTACCATGAGGACAAATATC-3′) and CB2 (5′-ATTACACCTCCTAATTTATTAGGAAT-3′) the regions of the *Cytb* gene in the polymerase chain reactions (PCR) amplification [39].

The PCR amplification was performed using Applied Biosystems ABI 3730 (Applied Biosystem, USA) in a 25 μL reaction mixture containing 12.5 μL of 2 × Taq PCR Master Mix (BBI), 1 μL of 10 μM forward primer, 1 μL of 10 μM reverse primer, 9.5 μL of ddH_2_O, and l μL of template DNA. The procedure for the PCR amplification was 4 min at 94℃, 35 cycles of 30 s at 94℃, 30 s at 48℃, and 1 min at 72℃, and a final extension for 10 min at 72℃. The reaction mixture without DNA template was included as negative control for each set of PCRs.

The PCR products were subjected to electrophoresis on a 1.5% agarose gel (UltraPure Agarose, Invitrogen) containing 10,000 × stock GelRed (Biotium) diluted at 1:10,000, visualized on a BioDoc-it imaging system (UVP), purified from the gel using ExoSAP-IT (USB, USA), and bidirectionally sequenced (using the above primers) on an ABI 3730XL Automated Sequencer using the BigDye Terminator Cycle Sequencing 3.1 Ready Reaction Kit (Applied Biosystems, USA).

### Data analysis

The forward and reverse sequences were assembled, aligned using ClustalW algorithm [40]. The obtained chromatograms were checked for the presence of ambiguous bases. The sequences were also translated to amino acids using the invertebrate mitochondrial code implemented in MEGA7 to check for the presence of stop codons and therefore pseudogenes [41]. The population genetic diversity was estimated using the program DnaSP 5.0 [42], as indexed by number of variable sites (*S*), parsimony informative sites, number of haplotypes (*Hn*), percentage (%) of haplotypes unique to a given geographical area, haplotype diversity (*Hd*), nucleotide diversity (*π*), and average number of nucleotide differences (*k*). To estimate the haplotype completeness a Coleman rarefaction curve was calculated with haploAccum of the spider package implemented in R software [43]. The Templeton, Crandall, and Sing (TCS) network of the haplotypes was performed using POPART [44, 45].

The population genetic structure was assessed with AMOVA in Arlequin3.5 according to the degree of differentiation between the regions (*F*_*CT*_), between the populations within the regions (*F*_*SC*_), and between all populations (*F*_*ST*_). The pairwise *F*_*ST*_ analyses among the populations and the regions were carried out with significance tests based on 1,000 permutations using Arlequin3.5 [46]. In order to test isolation by distance, the matrices of the genetic distance *F*_*ST*_/(1 − *F*_*ST*_) and the geographic distance (ln) between all 15 populations were compared using the Mantel test with 10,000 permutations [47] and the zt software package [48].

The historical demographic expansion was examined with Tajima's *D* and Fu’s *Fs* neutrality test and pairwise mismatch distribution [49–52], as implemented in Arlequin 3.5 [46]. Tajima's *D* and Fu’s *Fs* values are sensitive to demographic expansion, which usually leads to large negative values. Pairwise mismatch distributions were implemented to test whether a population experienced any expansion event. A goodness-of-fit test was used to determine the smoothness of the observed mismatch distribution (using Harpending’s raggedness index, Rag) and the degree of fit between the observed and simulated data (using the sum of squares deviation, SSD) [53, 54]. The expansion signal for a population was indicated by a smooth and unimodal distribution pattern with non-significant *p*-values for the SSD. The time of expansion was evaluated with the formula τ = 2μkt [52], where τ is the crest of mismatch distribution, μ is the nucleotide substitution rate, and k is the number of nucleotides.

## Supplementary Information

**Additional file 1: Table S1 **Geographical distribution of (A) *COI* and (B) *Cytb* haplotypes of *Strongyllodes variegatus *(Hap. = Haplotype; N = total number). **Table S2** Sample information of *Strongyllodes variegatus* (Fairmaire) specimens collected for the present study

**Additional file 2: Figure S1 **Individual-based rarefaction curves of haplotype diversity of *S variegatus* of in China.

**Additional file 3: Figure S2** Pairwise mismatch distributions of (a) *COI* and (b) *Cytb* genes for three derived regions. The x coordinate represents the number of pairwise differences among sequences, and the y coordinate represents the frequencies of pairwise differences in each region. The significance values (*p*) of the parameters were evaluated with 1,000 simulations; *P*_SSD_: *P *value for SSD (sum of squared deviations) *P*_R_: *P *value for Rag (Harpending’s raggedness index); τ: the index of population expansion.

## Data Availability

All mitochondrial and sample location data are available. DNA sequences are deposited at GenBank under the accession numbers [MN935027-MN935096 for *COI* haplotypes; MN935097–MF935163 for *Cytb* haplotypes].

## Abbreviations

mtDNA: Mitochondrial DNA
*COI*: Cytochrome *c* oxidase subunit I
*Cytb*: Cytochrome b
*Hd*: Haplotype diversity
*π*: Nucleotide diversity
*F*_*ST*_: Genetic differentiation
PCR: Polymerase chain reaction
AMOVA: Analysis of molecular variance
Rag: Harpending’s raggedness index
SSD: The sum of squares deviation

## References

[1] Borror DJ, De Long DM, Triplehorn CA (1981). An introduction to the study of insects (5th edition).

[2] He CG, Wang GL, Fan YH, Zou YX, Deng HY (1998). Study on a new pest *Strongyllodes Variegatus* (Fairmaire 1891) attacking rape. Acta Agric Boreali-occidentalis Sin.

[3] He CG (2001). Occurrence law and pesticide control of *Meligethes aeneus* (Coleoptera: Nitidulidae). Plant Protect.

[4] Hu BJ, Hou SM, Li CC, Zhou ZY, Zhang HS. Preliminary report of a new insect pest *Strongyllodes variegates* of oilseed rape in Anhui province. Proceedings of the 2012 Annual Conference of the Anhui Insect and Pathology Society. 2012;161–165.

[5] He CG, Pan F, Yang ZM, Han E, Fan YH, Zou YX (1996). Strategies and measures for integrated pest control in spring oilseed rape. Gansu Agric Sci Tech.

[6] Hou SM, Hu BC, Hu BJ, Li QS, Fei WX, Jiang YF, Fan ZX, Rong SB (2013). New pest *Strongyllodes variegatus* on winter oilseed rape in Anhui province. Chin J Oil Crop Sci.

[7] Zimmer CT, Maiwald F, Schorn C, Bass C, Ott MC, Nauen R (2014). A *de novo* transcriptome of European pollen beetle populations and its analysis with special reference to insecticide action and resistance: next-generation sequencing of the pollen beetle. Insect Mol Biol.

[8] Ouvrard P, Hicks DM, Mouland M, Nicholls JA, Baldock KC, Goddard MA, Kunin WE, Potts SG, Thieme T, Veromann E, Stone GN (2016). Molecular taxonomic analysis of the plant associations of adult pollen beetles (Nitidulidae: Meligethinae) and the population structure of *Brassicogethes aeneus*. Genome.

[9] Behura SK (2006). Molecular marker systems in insects: current trends and future avenues. Mol Ecol.

[10] Hurst GD, Jiggins FM (2005). Problems with mitochondrial DNA as a marker in population phylogeographic and phylogenetic studies: the effects of inherited symbionts. Proc R Soc Lond B Biol.

[11] Birungi J, Arctander P (2000). Large sequence divergence of mitochondrial DNA genotypes of the control region within populations of the African antelope kob (*Kobus kob*). Mol Ecol.

[12] Zanol J, Halanych KM, Struck TH, Fauchald K (2010). Phylogeny of the bristle worm family Eunicidae (Eunicida Annelida) and the phylogenetic utility of noncongruent 16S COI and 18S in combined analyses. Mol Phylogenet Evol.

[13] Men Q, Xue G, Mu D, Hu Q, Huang M (2017). Mitochondrial DNA markers reveal high genetic diversity and strong genetic differentiation in populations of *Dendrolimus kikuchii* Matsumura (Lepidoptera: Lasiocampidae). PLoS ONE.

[14] Meng XF, Shi M, Chen XX (2008). Population genetic structure of *Chilo suppressalis* (Walker) (Lepidoptera: Crambidae): strong subdivision in China inferred from microsatellite markers and mtDNA gene sequences. Mol Ecol.

[15] Du Z, Liu H, Li H, Ishikawa T, Su ZH, Cai WZ, Kamitani S, Tadauchi O (2018). Invasion of the assassin bug *Agriosphodrus dohrni* (Hemiptera: Reduviidae) to Japan: source estimation inferred from mitochondrial and nuclear gene sequences. Int J Biol Macromol.

[16] Cesari M, Maistrello L, Ganzerli F, Dioli P, Rebecchi L, Guidetti R (2015). A pest alien invasion in progress: potential pathways of origin of the brown marmorated stink bug *Halyomorpha halys* populations in Italy. J Pest Sci.

[17] Gariepy TD, Haye T, Fraser H, Zhang J (2014). Occurrence genetic diversity and potential pathways of entry of *Halyomorpha halys* in newly invaded areas of Canada and Switzerland. J Pest Sci.

[18] Valentin RE, Nielsen AL, Wiman NG, Lee DH (2017). Fonseca DM, Global invasion network of the brown marmorated stink bug *Halyomorpha halys*. Sci Rep.

[19] Meng XF, Shi M, Chen XX (2008). Population genetic structure of *Chilo suppressalis* (Walker) (Lepidoptera: Crambidae): strong subdivision in China inferred from microsatellite markers and mtDNA gene sequences. Mol Ecol.

[20] Ruedi M, Walter S, Fischer MC, Scaravelli D, Excoffier L, Heckel G (2008). Italy as a major Ice Age refuge area for the bat *Myotis myotis* (Chiroptera: Vespertilionidae) in Europe. Mol Ecol.

[21] Razgour O, Juste J, Ibáñez C, Kiefer A, Rebelo H, Puechmaille SJ, Arlettaz R, Burke T, Dawson DA, Beaumont M, Jones G (2013). The shaping of genetic variation in edge-of-range populations under past and future climate change. Ecol Lett.

[22] Chen MH, Dorn S (2009). Microsatellites reveal genetic differentiation among populations in an insect species with high genetic variability in dispersal, the codling moth, *Cydia pomonella* (L.) (Lepidoptera: Tortricidae). Bull Entomol Res..

[23] Krantz DE, Williams DF, Jones DS (1987). Ecological and paleoenvironmental information using stable isotope profiles from living and fossil molluscs. Palaeogeogr Palaeoclim Palaeoecol.

[24] Peterson MA, Denno RF (1998). The influence of dispersal and diet breadth on patterns of genetic isolation by distance in phytophagous insects. Am Nat.

[25] Roff DA (1994). The evolution of flightlessness: is history important?. Evol Ecol.

[26] Juhel AS, Barbu CM, Valantin-Morison M, Gauffre B, Leblois R, Olivares J, Franck P (2019). Limited genetic structure and demographic expansion of the *Brassicogethes aeneus* populations in France and in Europe. Pest Manag Sci.

[27] Kazachkova N, Meijer J, Ekbom B (2007). Genetic diversity in pollen beetles (*Meligethes aeneus*) in Sweden: role of spatial temporal and insecticide resistance factors. Agric For Entomol.

[28] Kazachkova N, Meijer J, Ekbom B (2008). Genetic diversity in European pollen beetle *Meligethes aeneus* (Coleoptera: Nitidulidae) populations assessed using AFLP analysis. Eur J Entomol.

[29] Mauchline AL, Cook SM, Powell W, Chapman JW, Osborne JL (2017). Migratory flight behaviour of the pollen beetle *Meligethes aeneus*. Pest Manag Sci..

[30] Williams IH (2010). The major insect pests of oilseed rape in Europe and their management an overview. Biocontrol-based integrated management of oilseed rape pests.

[31] Endersby NM, McKechnie SW, Ridland PM, Weeks AR (2006). Microsatellites reveal a lack of structure in Australian populations of the diamondback moth *Plutella xylostella* (L.). Mol Ecol..

[32] Brower AVZ (1994). Rapid morphological radiation and convergence among races of the butterfly *Heliconius erato* inferred from patterns of mitochondrial DNA evolution. Proc Natl Acad Sci USA.

[33] Shi YF, Cui ZJ, Su Z (2006). The quaternary glaciations and environ-mental variations in China.

[34] Deng LL (2006). The Pleistocene climate of Changjiang valley. J Guizhou Normal Univ (Natural Sciences).

[35] Hewitt G (2000). The genetic legacy of the Quaternary ice ages. Nature.

[36] Smith CI, Farrell BD (2005). Range expansions in the flightless longhorn cactus beetles *Moneilema gigas* and *Moneilema armatum* in response to Pleistocene climate changes. Mol Ecol.

[37] Tzedakis PC, Roucoux KH, de Abreu L, Shackleton NJ (2004). The duration of forest stages in southern Europe and interglacial climate variability. Science.

[38] Ayres RM, Pettigrove VJ, Hoffmann AA (2010). Low diversity and high levels of population genetic structuring in intro duced eastern mosquitofish (*Gambusia holbrooki*) in the greater Melbourne area, Australia. Biol Invasions.

[39] Simon C, Frati F, Beckenbach A, Crespi B, Liu H, Flook P (1994). Evolution weighting and phylogenetic utility of mitochondrial gene sequences and a compilation of conserved polymerase chain reaction primers. Ann Entomol Soc Am.

[40] Thompson JD, Higgins DG, Gibson TJ (1994). CLUSTAL W: improving the sensitivity of progressive multiple sequence alignment through sequence weighting position-specific gap penalties and weight matrix choice. Nucleic Acids Res.

[41] Kumar S, Stecher G, Tamura K (2016). MEGA7: molecular evolutionary genetics analysis version 7.0 for bigger datasets. Mol Biol Evol..

[42] Librado P, Rozas J (2009). DnaSP v5: a software for comprehensive analysis of DNA polymorphism data. Bioinformatics.

[43] Gotelli NJ, Colwell RK (2001). Quantifying biodiversity: procedures and pitfalls in the measurement and comparison of species richness. Ecol Lett.

[44] Templeton AR, Crandall KA, Sing CF (1992). A cladistic analysis of phenotypic associations with haplotypes inferred from restriction endonuclease mapping and DNA sequence data III Cladogram estimation. Genetics.

[45] Leigh JW, Bryant D (2015). POPART: full-feature software for haplotype network construction. Methods Ecol Evol.

[46] Excoffier L, Lischer HE (2010). Arlequin suite ver 3.5: a new series of programs to perform population genetics analyses under Linux and Windows. Mol Ecol Resour..

[47] Mantel N (1967). The detection of disease clustering and a generalized regression approach. Cancer Res.

[48] Bonnet E, Van de Peer Y (2002). zt: a sofware tool for simple and partial mantel tests. J Stat Softw.

[49] Tajima F (1989). The effect of change in population size on DNA polymorphism. Genetics.

[50] Fu YX (1997). Statistical tests of neutrality of mutations against population growth hitchhiking and background selection. Genetics.

[51] Rogers AR, Harpending H (1992). Population growth makes waves in the distribution of pair-wise genetic differences. Mol Biol Evol.

[52] Slatkin M, Hudson RR (1991). Pairwise comparisons of mitochondrial DNA sequences in stable and exponentially growing populations. Genetics.

[53] Harpending HC (1994). Signature of ancient population growth in a low-resolution mitochondrial DNA mismatch distribution. Hum Biol.

[54] Schneider S, Excoffier L (1999). Estimation of past demographic parameters from the distribution of pairwise differences when the mutation rates vary among sites: application to human mitochondrial DNA. Genetics.

