# Supplementary material for: High genetic diversity and strong genetic structure of Strongyllodes variegatus populations in oilseed rape production areas of China
Source: BMC Ecol Evol. 2021 Feb 9;21:18. doi: 10.1186/s12862-021-01752-6 (PMC7871595; doi:10.1186/s12862-021-01752-6)
Supplement: Supplementary file 1 — Additional file 1: Table S1 Geographical distribution of (A) COI and (B) Cytb haplotypes of Strongyllodes variegatus (Hap. = Haplotype; N = total number). Table S2 Sample information of Strongyllodes variegatus (Fairmaire) specimens collected for the present study [file 12862_2021_1752_MOESM1_ESM.docx]

**Additional Files**

**Additional file 1: Table S1** Geographical distribution of (A) *COI* and (B) *Cytb* haplotypes of *Strongyllodes variegates* (Hap. = Haplotype; N = total number)

| (A) Hap. | GDQH | HZGS | ZYGS | GYSC | HZSX | AKSX | FJCQ | ESHB | LCHB | AQAH | LAAH | HFAH | CHAH | NJJS | ZJJS | N |
| --- | --- | --- | --- | --- | --- | --- | --- | --- | --- | --- | --- | --- | --- | --- | --- | --- |
| H1 |  | 1 | 3 | 3 | 3 | 3 |  |  | 1 | 24 | 16 | 21 | 18 | 22 | 17 | 132 |
| H2 |  |  |  | 11 | 8 | 12 | 10 | 3 | 15 |  |  |  |  |  |  | 59 |
| H3 |  |  |  |  |  |  |  |  |  | 3 |  | 8 | 2 | 7 | 9 | 29 |
| H4 | 14 | 18 | 14 |  | 3 | 6 | 2 |  | 3 |  |  |  |  |  |  | 60 |
| H5 | 7 | 2 |  |  |  |  |  |  | 1 |  |  |  |  |  |  | 10 |
| H6 | 1 |  |  |  | 1 |  |  |  |  |  |  |  |  |  |  | 2 |
| H7 |  | 2 |  |  | 2 | 2 |  |  |  |  |  |  |  |  |  | 6 |
| H8 |  |  | 1 |  |  | 2 |  |  |  |  |  |  |  |  |  | 3 |
| H9 |  |  | 2 | 1 |  | 1 |  |  |  |  |  |  |  |  |  | 4 |
| H10 | 11 | 5 |  |  |  |  |  |  |  |  |  |  |  |  |  | 16 |
| H11 | 1 |  |  |  |  |  |  |  |  |  |  |  |  |  |  | 1 |
| H12 |  | 2 |  |  |  |  |  |  |  |  |  |  |  |  |  | 2 |
| H13 |  | 1 |  |  |  |  |  |  |  |  |  |  |  |  |  | 1 |
| H14 |  | 1 |  |  |  |  |  |  |  |  |  |  |  |  |  | 1 |
| H15 |  | 2 |  |  |  |  |  |  |  |  |  |  |  |  |  | 2 |
| H16 |  |  | 2 |  |  |  |  |  |  |  |  |  |  |  |  | 2 |
| H17 |  |  | 2 |  |  |  |  |  |  |  |  |  |  |  |  | 2 |
| H18 |  |  |  | 1 |  |  | 1 |  |  |  |  |  |  |  |  | 2 |
| H19 |  |  |  | 1 | 1 |  |  |  |  |  |  |  |  |  |  | 2 |
| H20 |  |  |  | 1 | 2 |  |  |  |  |  |  |  |  |  |  | 3 |
| H21 |  |  |  | 2 | 2 |  | 1 |  |  |  |  |  |  |  |  | 5 |
| H22 |  |  |  | 1 |  |  |  |  |  |  |  |  |  |  |  | 1 |
| H23 |  |  |  | 3 | 1 |  |  |  | 3 |  |  |  |  |  |  | 7 |
| H24 |  |  |  | 2 |  |  |  |  |  |  |  |  |  |  |  | 2 |
| H25 |  |  |  | 1 |  |  |  |  |  |  |  |  |  |  |  | 1 |
| H26 |  |  |  | 1 |  |  |  |  | 4 |  |  |  |  |  |  | 5 |
| H27 |  |  |  | 1 |  |  |  |  |  |  |  |  |  |  |  | 1 |
| H28 |  |  |  | 1 |  |  |  |  |  |  |  |  |  |  |  | 1 |
| H29 |  |  |  |  | 1 | 1 |  |  |  |  |  |  |  |  |  | 2 |
| H30 |  |  |  |  | 2 | 2 |  | 1 |  |  |  |  |  |  |  | 5 |
| H31 |  |  |  |  | 1 |  | 1 | 1 | 1 |  |  |  |  |  |  | 4 |
| H32 |  |  |  |  | 1 |  |  |  |  |  |  |  |  |  |  | 1 |
| H33 |  |  |  |  | 1 |  |  |  |  |  |  |  |  |  |  | 1 |
| H34 |  |  |  |  | 1 |  |  |  |  |  |  |  |  |  |  | 1 |
| H35 |  |  |  |  |  | 1 |  |  |  |  |  |  |  |  |  | 1 |
| H36 |  |  |  |  |  | 1 |  |  |  |  |  |  |  |  |  | 1 |
| H37 |  |  |  |  |  | 2 | 2 |  | 1 |  |  |  |  |  |  | 5 |
| H38 |  |  |  |  |  | 1 |  |  |  |  |  |  |  |  |  | 1 |
| H39 |  |  |  |  |  | 1 |  |  |  |  |  |  |  |  |  | 1 |
| H40 |  |  |  |  |  |  | 1 |  |  |  |  |  |  |  |  | 1 |
| H41 |  |  |  |  |  |  | 6 |  |  |  |  |  |  |  |  | 6 |
| H42 |  |  |  |  |  |  | 1 |  |  |  |  |  |  |  |  | 1 |
| H43 |  |  |  |  |  |  | 1 |  |  |  |  |  |  |  |  | 1 |
| H44 |  |  |  |  |  |  | 1 |  |  |  |  |  |  |  |  | 1 |
| H45 |  |  |  |  |  |  | 1 |  |  |  |  |  |  |  |  | 1 |
| H46 |  |  |  |  |  |  | 1 |  |  |  |  |  |  |  |  | 1 |
| H47 |  |  |  |  |  |  | 1 |  | 1 |  |  |  |  |  |  | 2 |
| H48 |  |  |  |  |  |  |  | 1 |  |  |  |  |  |  |  | 1 |
| H49 |  |  |  |  |  |  |  | 1 |  |  |  |  |  |  |  | 1 |
| H50 |  |  |  |  |  |  |  | 1 |  |  |  |  |  |  |  | 1 |
| H51 |  |  |  |  |  |  |  |  | 1 |  |  |  |  |  |  | 1 |
| H52 |  |  |  |  |  |  |  |  | 1 |  |  |  |  |  |  | 1 |
| H53 |  |  |  |  |  |  |  |  |  | 1 | 1 |  | 2 | 1 | 2 | 7 |
| H54 |  |  |  |  |  |  |  |  |  | 1 | 2 | 1 | 1 |  |  | 5 |
| H55 |  |  |  |  |  |  |  |  |  | 1 | 1 |  |  |  |  | 2 |
| H56 |  |  |  |  |  |  |  |  |  | 1 |  |  |  |  |  | 1 |
| H57 |  |  |  |  |  |  |  |  |  | 1 |  |  |  |  |  | 1 |
| H58 |  |  |  |  |  |  |  |  |  | 1 |  |  |  |  |  | 1 |
| H59 |  |  |  |  |  |  |  |  |  | 1 |  |  |  |  |  | 1 |
| H60 |  |  |  |  |  |  |  |  |  | 1 |  |  |  |  |  | 1 |
| H61 |  |  |  |  |  |  |  |  |  | 2 |  |  |  |  |  | 2 |
| H62 |  |  |  |  |  |  |  |  |  |  | 1 |  |  |  |  | 1 |
| H63 |  |  |  |  |  |  |  |  |  |  |  | 2 |  |  |  | 2 |
| H64 |  |  |  |  |  |  |  |  |  |  |  | 1 |  |  |  | 1 |
| H65 |  |  |  |  |  |  |  |  |  |  |  | 1 |  |  |  | 1 |
| H66 |  |  |  |  |  |  |  |  |  |  |  |  | 1 |  | 1 | 2 |
| H67 |  |  |  |  |  |  |  |  |  |  |  |  | 2 |  |  | 2 |
| H68 |  |  |  |  |  |  |  |  |  |  |  |  |  | 1 |  | 1 |
| H69 |  |  |  |  |  |  |  |  |  |  |  |  |  |  | 1 | 1 |
| H70 |  |  |  |  |  |  |  |  |  |  |  |  |  |  | 1 | 1 |
| (B) Hap. | GDQH | HZGS | ZYGS | GYSC | HZSX | AKSX | FJCQ | ESHB | LCHB | AQAH | LAAH | HFAH | CHAH | NJJS | ZJJS | N |
| H1 | 12 | 24 | 15 | 3 | 11 | 10 | 3 |  | 3 | 18 | 6 | 13 | 13 | 12 | 15 | 158 |
| H2 |  |  |  | 9 | 5 | 12 | 13 | 6 | 16 |  |  |  |  |  |  | 61 |
| H3 |  |  |  |  |  |  |  |  |  | 8 | 9 | 9 | 5 | 8 | 9 | 48 |
| H4 |  | 1 |  |  | 2 | 2 |  |  |  | 1 |  | 3 |  |  |  | 9 |
| H5 | 1 |  |  |  |  |  |  |  |  | 1 |  |  | 1 |  |  | 3 |
| H6 |  |  | 4 |  |  |  |  |  |  | 1 |  |  |  |  |  | 5 |
| H7 |  |  | 2 | 1 |  |  |  |  |  |  |  |  |  |  |  | 3 |
| H8 |  |  |  | 1 |  |  |  |  |  |  |  | 1 | 1 |  |  | 3 |
| H9 |  |  |  | 1 | 1 |  | 4 |  |  |  |  | 2 |  |  | 1 | 9 |
| H10 | 14 | 7 |  |  |  |  |  |  |  |  |  |  |  |  |  | 21 |
| H11 | 4 | 2 |  |  |  |  |  |  |  |  |  |  |  |  |  | 6 |
| H12 | 2 |  |  |  |  |  |  |  |  |  |  |  |  |  |  | 2 |
| H13 | 1 |  | 3 |  |  |  |  |  |  |  |  |  |  |  |  | 4 |
| H14 |  |  |  | 1 |  | 1 |  |  |  |  |  |  |  |  |  | 2 |
| H15 |  |  |  | 2 | 1 |  | 1 |  |  |  |  |  |  |  |  | 4 |
| H16 |  |  |  | 5 | 4 |  |  |  |  |  |  |  |  |  |  | 9 |
| H17 |  |  |  | 2 |  |  |  |  |  |  |  |  |  |  |  | 2 |
| H18 |  |  |  | 1 |  |  |  |  |  |  |  |  |  |  |  | 1 |
| H19 |  |  |  | 1 |  |  |  |  |  |  |  |  |  |  |  | 1 |
| H20 |  |  |  | 1 |  |  |  |  | 1 |  |  |  |  |  |  | 2 |
| H21 |  |  |  | 1 |  |  |  |  |  |  |  |  |  |  |  | 1 |
| H22 |  |  |  | 1 |  |  |  |  |  |  |  |  |  |  |  | 1 |
| H23 |  |  |  |  | 1 |  | 1 |  |  |  |  |  |  |  |  | 2 |
| H24 |  |  |  |  | 1 |  |  |  |  |  |  |  |  |  |  | 1 |
| H25 |  |  |  |  | 1 |  |  |  |  |  |  |  |  |  |  | 1 |
| H26 |  |  |  |  | 2 |  |  |  |  |  |  |  |  |  |  | 2 |
| H27 |  |  |  |  | 1 |  |  |  |  |  |  |  |  |  |  | 1 |
| H28 |  |  |  |  |  | 1 |  |  |  |  |  |  |  |  |  | 1 |
| H29 |  |  |  |  |  | 1 |  |  |  |  |  |  |  |  |  | 1 |
| H30 |  |  |  |  |  | 1 | 2 |  |  |  |  |  |  |  |  | 3 |
| H31 |  |  |  |  |  | 2 | 3 |  |  |  |  |  |  |  |  | 5 |
| H32 |  |  |  |  |  | 1 |  |  |  |  |  |  |  |  |  | 1 |
| H33 |  |  |  |  |  | 1 |  |  |  |  |  |  |  |  |  | 1 |
| H34 |  |  |  |  |  | 1 | 1 |  |  |  |  |  |  |  |  | 2 |
| H35 |  |  |  |  |  | 1 |  |  |  |  |  |  |  |  |  | 1 |
| H36 |  |  |  |  |  | 1 |  |  |  |  |  |  |  |  |  | 1 |
| H37 |  |  |  |  |  |  | 1 | 1 | 1 |  |  |  |  |  |  | 3 |
| H38 |  |  |  |  |  |  | 1 |  |  |  |  |  |  |  |  | 1 |
| H39 |  |  |  |  |  |  |  | 1 |  |  |  |  |  |  |  | 1 |
| H40 |  |  |  |  |  |  |  |  | 1 |  |  |  |  |  |  | 1 |
| H41 |  |  |  |  |  |  |  |  | 1 |  |  |  |  |  |  | 1 |
| H42 |  |  |  |  |  |  |  |  | 1 |  |  |  |  |  |  | 1 |
| H43 |  |  |  |  |  |  |  |  | 1 |  |  |  |  |  |  | 1 |
| H44 |  |  |  |  |  |  |  |  | 1 |  |  |  |  |  |  | 1 |
| H45 |  |  |  |  |  |  |  |  | 1 |  |  |  |  |  |  | 1 |
| H46 |  |  |  |  |  |  |  |  | 1 |  |  |  |  |  |  | 1 |
| H47 |  |  |  |  |  |  |  |  | 1 |  |  |  |  |  |  | 1 |
| H48 |  |  |  |  |  |  |  |  | 1 |  |  |  |  |  |  | 1 |
| H49 |  |  |  |  |  |  |  |  | 1 |  |  |  |  |  |  | 1 |
| H50 |  |  |  |  |  |  |  |  | 1 |  |  |  |  |  |  | 1 |
| H51 |  |  |  |  |  |  |  |  |  | 2 |  |  | 1 | 1 | 1 | 5 |
| H52 |  |  |  |  |  |  |  |  |  | 4 | 5 | 3 | 1 |  |  | 13 |
| H53 |  |  |  |  |  |  |  |  |  | 1 |  |  |  |  |  | 1 |
| H54 |  |  |  |  |  |  |  |  |  | 1 |  |  |  |  |  | 1 |
| H55 |  |  |  |  |  |  |  |  |  |  | 1 | 1 |  |  |  | 2 |
| H56 |  |  |  |  |  |  |  |  |  |  |  | 1 |  | 1 |  | 2 |
| H57 |  |  |  |  |  |  |  |  |  |  |  | 1 |  |  |  | 1 |
| H58 |  |  |  |  |  |  |  |  |  |  |  |  | 2 | 1 |  | 3 |
| H59 |  |  |  |  |  |  |  |  |  |  |  |  | 1 |  |  | 1 |
| H60 |  |  |  |  |  |  |  |  |  |  |  |  | 1 |  |  | 1 |
| H61 |  |  |  |  |  |  |  |  |  |  |  |  |  | 5 |  | 5 |
| H62 |  |  |  |  |  |  |  |  |  |  |  |  |  | 1 | 1 | 2 |
| H63 |  |  |  |  |  |  |  |  |  |  |  |  |  | 1 | 1 | 2 |
| H64 |  |  |  |  |  |  |  |  |  |  |  |  |  | 1 |  | 1 |
| H65 |  |  |  |  |  |  |  |  |  |  |  |  |  |  | 1 | 1 |
| H66 |  |  |  |  |  |  |  |  |  |  |  |  |  |  | 1 | 1 |
| H67 |  |  |  |  |  |  |  |  |  |  |  |  |  |  | 1 | 1 |

**Additional file 2: Figure S1** Individual-based rarefaction curves of haplotype diversity of *S variegatus* of in China.

**Additional file 3: Figure S2** Pairwise mismatch distributions of (a) *COI* and (b) *Cytb* genes for three derived regions. The x coordinate represents the number of pairwise differences among sequences, and the y coordinate represents the frequencies of pairwise differences in each region. The significance values (*p*) of the parameters were evaluated with 1,000 simulations; *P*_SSD_: *P* value for SSD (sum of squared deviations) *P*_R_: *P* value for Rag (Harpending’s raggedness index); τ: the index of population expansion.

**Additional file 4: Table S2** Sample information of *Strongyllodes variegatus* (Fairmaire) specimens collected for the present study

| Province | Location | Abbreviation | Longitude | Latitude | Years | Sample size |
| --- | --- | --- | --- | --- | --- | --- |
| Qinghai | Guide | GDQH | 101.43 | 36.05 | 2012 | 34 |
| Gansu | Hezheng | HZGS | 103.35 | 35.43 | 2013, 2015 | 34 |
|  | Zhenyuan | ZYGS | 107.32 | 35.53 | 2019 | 24 |
| Sichuang | Guangyuan | GYSC | 105.79 | 32.59 | 2015 | 30 |
| Shaanxi | Hanzhong | HZSX | 106.67 | 33.16 | 2015 | 30 |
|  | Ankang | AKSX | 108.25 | 32.05 | 2015, 2017 | 35 |
| Chongqing | Fengjie | FJCQ | 109.44 | 31.01 | 2015 | 30 |
| Hubei | Enshi | ESHB | 109.72 | 30.61 | 2015 | 8 |
|  | Lichuang | LCHB | 108.77 | 30.48 | 2019 | 32 |
| Anhui | Anqing | AQAH | 116.58 | 30.63 | 2016, 2017 | 37 |
|  | Liu'an | LAAH | 116.70 | 31.79 | 2016, 2017 | 21 |
|  | Hefei | HFAH | 117.23 | 31.88 | 2015, 2016, 2017 | 34 |
|  | Caohu | CHAH | 117.89 | 31.62 | 2012, 2015 | 26 |
| Jiangsu | Nanjing | NJJS | 118.47 | 32.05 | 2019 | 31 |
|  | Zhenjiang | ZJJS | 119.18 | 31.94 | 2015 | 31 |
