# Supplementary figures and images for: High genetic diversity and strong genetic structure of Strongyllodes variegatus populations in oilseed rape production areas of China
Source: BMC Ecol Evol. 2021 Feb 9;21:18. doi: 10.1186/s12862-021-01752-6 (PMC7871595; doi:10.1186/s12862-021-01752-6)

random method of haplotype accumulation

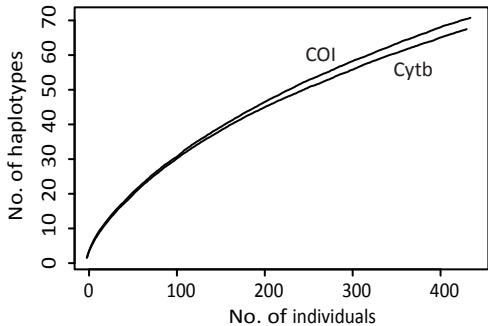

Supplement: Supplementary file 2 — Additional file 2: Figure S1 Individual-based rarefaction curves of haplotype diversity of S variegatus of in China. [file 12862_2021_1752_MOESM2_ESM.pdf]

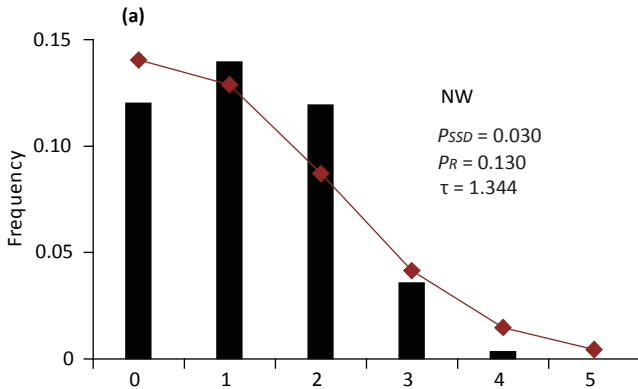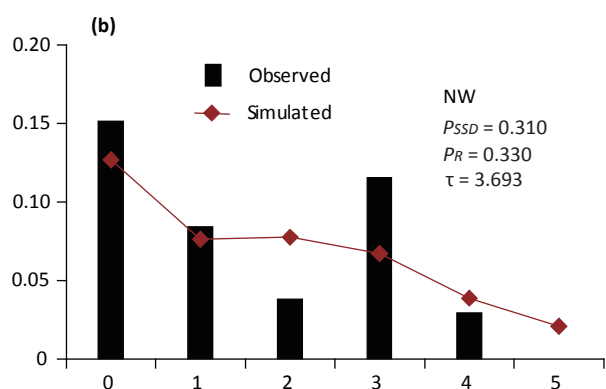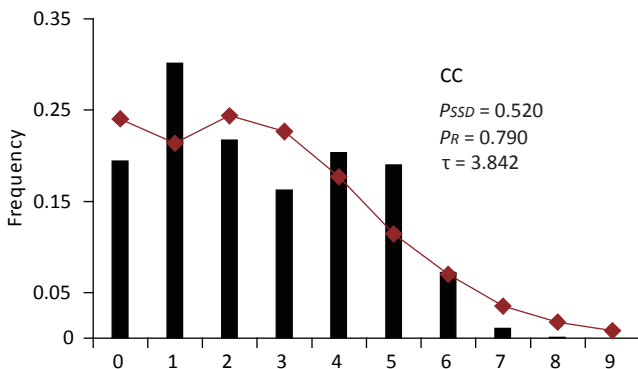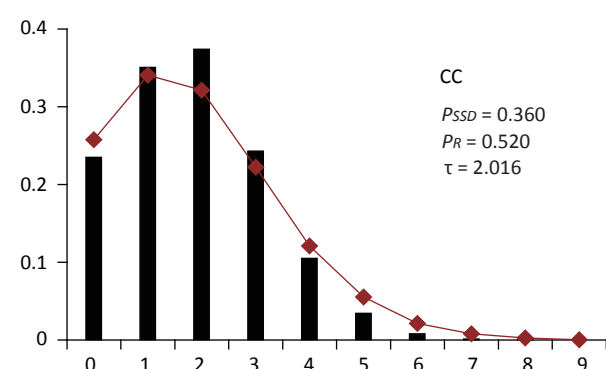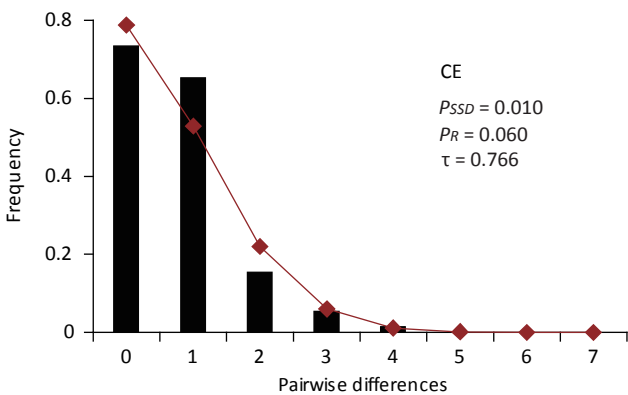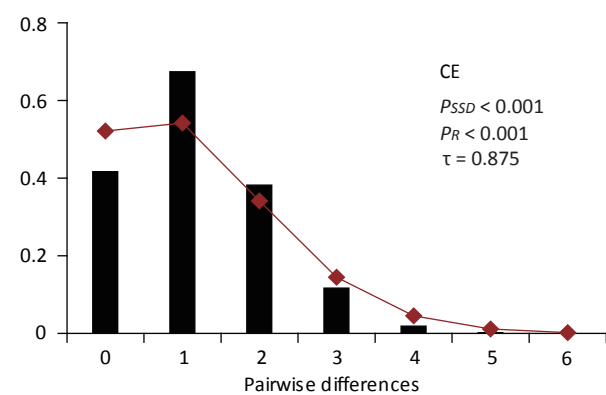

Supplement: Supplementary file 3 — Additional file 3: Figure S2 Pairwise mismatch distributions of (a) COI and (b) Cytb genes for three derived regions. The x coordinate represents the number of pairwise differences among sequences, and the y coordinate represents the frequencies of pairwise differences in each region. The significance values (p) of the parameters were evaluated with 1,000 simulations; PSSD: P value for SSD (sum of squared deviations) PR: P value for Rag (Harpending’s raggedness index); τ: the index of population expansion. [file 12862_2021_1752_MOESM3_ESM.pdf]
